# Influence of Differences in the Density of Seawater on the Measurement of the Underwater Gravity Gradient

**DOI:** 10.3390/s23020714

**Published:** 2023-01-08

**Authors:** Pengfei Xian, Bing Ji, Shaofeng Bian, Jingwen Zong, Tao Zhang

**Affiliations:** Department of Navigation, Naval University of Engineering, Wuhan 430033, China

**Keywords:** gravity gradient, seawater density, right rectangular prism, forward modeling

## Abstract

In preparing gravity gradient reference maps for navigation purposes, researchers have tended to use a constant value for the density of seawater. However, the actual seawater density at a particular location may vary due to the effects of longitude, latitude and bathymetry. In this study, the right rectangular prism method was used to calculate the disturbing gravity gradient caused by the mass deficiency of seawater for three different seawater profiles in an area east of Taiwan. For this purpose, two seawater density models were used as alternatives to the constant seawater density model, and the alteration in the gravity gradient was calculated to quantify the error in the gravity gradient as a result of using a constant seawater density. The results demonstrated that the error in the gravity gradient can reach 1E for water at large depths. Moreover, the difference between the amplitude of the error of the corrected thermocline and that for the uncorrected seawater density model was found to be quite small. If a gravity gradient reference map with accuracy better than 1E is to be realized, the seawater density cannot be taken as constant during forward modeling.

## 1. Introduction

In oceanography, the parameters of the gravity field depend on the geology, topography, the density of seawater and other factors. In the process of gravity gradient matching navigation, the best matching position is determined by comparing the gravity gradient signal measured in real-time with the gravity gradient reference map. When using the gravity gradient reference map prepared from the digital elevation model, the crustal density is usually regarded as a constant [1,2,3]. The prism method with constant density has always been an effective tool in geophysics, but in many geological environments, this simple density model may not be valid. For example, the density of sedimentary basins usually increases exponentially with depth, and the geological structure of sedimentary basins may be complicated by other geological processes. Due to the different geology, the rock density can vary from about 2.0 g/cm^3^ to 3.0 g/cm^3^. A hybrid algorithm was proposed as a mean for inverting surface gravity anomalies to attain the subsurface density distribution [4]. It has been found that the influence of the error in underground density for the forward modeling of the gravity gradient cannot be ignored. When the error in the density is only 0.07 g/cm^3^, the alteration in the generated gravity gradient reaches 1E, and when the error is 0.13 g/cm^3^, the alteration in the gravity gradient reaches about 10E [5,6]. Therefore, researchers have adopted methods such as the isostasy model to reduce the errors caused by the difference of the crustal density [5,7,8]. Roland et al. described the density distribution, which varied with depth, using polynomials and derived the gravitational potential and its first and second derivatives of the rectangular prism with indefinite density [9]. In the case of ocean water, as the density of seawater is less than that of the crust, the gravitational effect includes the mass deficiency of seawater relative to the land. Therefore, the seawater mass deficiency, the marine topography and the marine geology should be taken into account in the forward modeling of the gravity gradient field. Tenzer et al. used spherical harmonic analysis and gravity field synthesis to calculate the gravitational field generated by the seawater globally [10,11,12,13]. Wang and Yang calculated the gravity gradient of an ocean area [14,15,16]. Xian discussed the contribution of the sea level anomaly to the gravity gradient [17]. In the aforementioned studies, the density of the seawater is considered as a constant. However, the actual density of seawater is not constant. If the actual distribution of the density of seawater is approximated to an average value, the relative inaccuracy of the forward modeling results of the gravitational field of the ocean would be as high as about 2%, and the calculated errors corresponding to the gravitational potential and gravity would be 550 m^2^/s^2^ and 15 mGal, respectively [13]. The seawater density model, as proposed by Gladkikh et al. (2011), was used in this study to calculate the gravity gradient, which was used to quantify the error in the gravity gradient caused by using seawater of constant density.

## 2. Data and Methods

The seawater density data are from the World Oceanographic Atlas (WOA18), which is based on the profile data of the World Oceanographic Database (WOD). It contains data for the mean field grids at a longitude and latitude of 1/4° and 1° for the temperature, salinity, oxygen, phosphate, silicate, and nitrate via objective monitoring, analyses and quality control programs. The database can be used to create boundary or initial conditions for various ocean models, verify numerical simulation of the oceans, and verify satellite data. The temperature and salinity data of this dataset are divided into monthly, quarterly, and annual long-term monthly average data, and include seven time periods: 1955–1964, 1965–1974, 1975–1984, 1985–1994, 1995–2004, 2005–2017 and 1981–2010. The horizontal direction covers the global ocean, and the vertical direction is divided into 102 layers according to the standard layer [18,19].

The topographic data using the GEBCO_2021 global grid bathymetric dataset, released in July 2021, is a global ocean and land terrain model. The elevation data in meters is provided on a 15 arc second geographic longitude and latitude grid. The seawater bathymetry is negative, and the terrain height is positive. The GEBCO grid data are constructed based on data from multiple sources, including regional global grids and hundreds of surveys provided by international and national databases and industrial partners such as IHO-DCDB.

Gravity gradient forward modeling is based on Newton’s law of gravity, using known mass anomalies to calculate the gravity gradient. The calculation methods used previously in the forward modeling of gravity gradient using the terrain mainly include the right rectangular prism, the polyhedron, direct numerical integration, and fast Fourier transform methods. By comparing the results for the various methods, the results of the right rectangular prism method have been demonstrated to yield the highest accuracy [1,3]; thus, this method has been adopted in this study for calculation purposes.

## 3. Seawater Density Distribution

The density of seawater is affected directly by the temperature of seawater (due to thermal expansion; the density decreases with the increase in temperature), the salinity (due to an increase in solute concentration; density increases with increase in salinity) and pressure (density increases with increase in pressure). The actual density of seawater varies between 1020 and 1050 kg/m^3^, with the largest contribution being due to the variability in pressure [20]. Surface runoff and glacial meltwater will also indirectly affect the seawater density by affecting the temperature and salinity [21]. In oceanography, only the last two digits of the density values are usually used in the literature for convenience.

**Figure 1:**
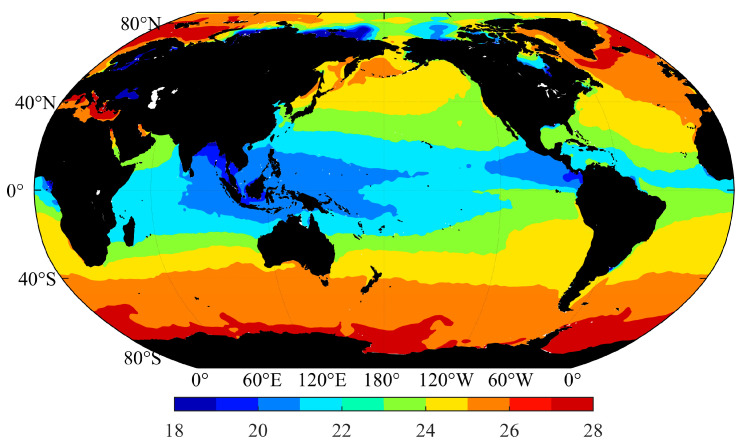
Global surface seawater density (unit: kg/m^3^).

The density of surface seawater is greatly affected by the temperature of the seawater, as shown in Figure 1, which is generally distributed in the latitudinal direction. The highest temperature and the lowest salinity lead to the lowest density occurring in surface waters at the equator, and the density gradually increases from 1020 kg/m^3^ at the equator to 1029 kg/m^3^ in the polar region. In some continental coasts and Arctic regions, low density areas will appear due to inputs from the land source such as surface runoff and glacial meltwater. Given that the range in the variation of the temperature of the surface seawater is larger than the range in the variation of the salinity, the influence of the temperature of surface seawater on density is greater than that of salinity. In polar regions, the influence of salinity on density is highlighted due to the low temperature of seawater.

**Figure 2:**
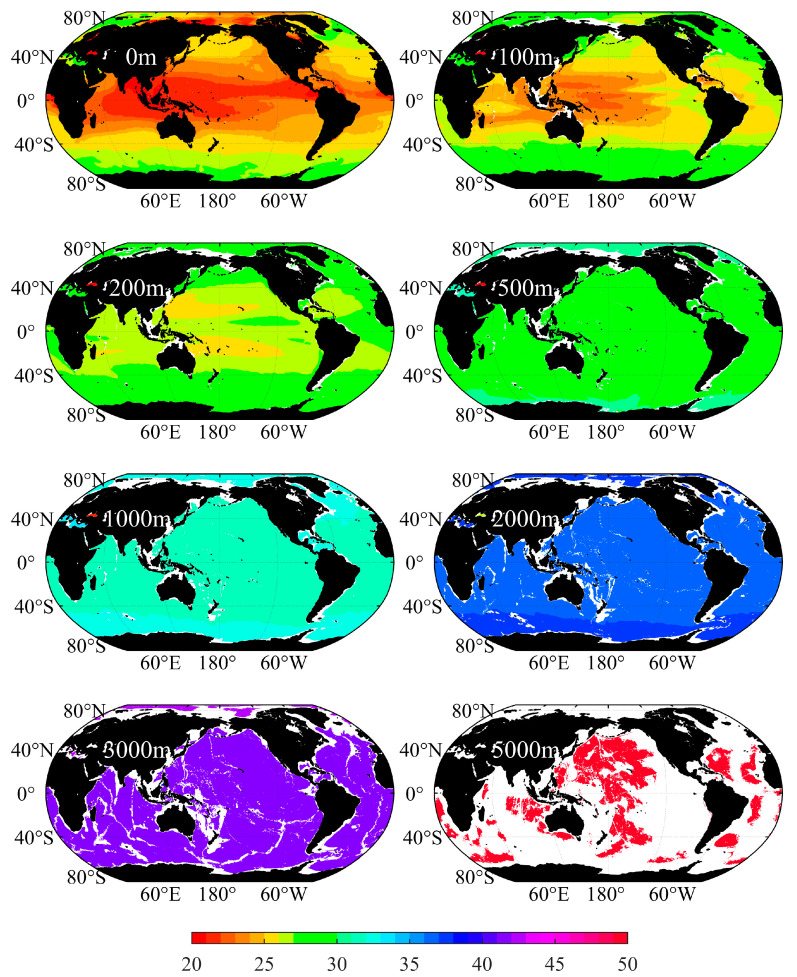
Density of seawater at different depths in the global ocean (unit: kg/m^3^).

Compared with the upper layers of seawater, the density of deep seawater hardly changes with latitude, as shown in Figure 2. The difference of global seawater density at different locations within 200 m can be more than 20 kg/m^3^. At the eastern boundary of the ocean, the process of upwelling transports seawater at a lower temperature in the deep layers to the upper layers, making the seawater density at the eastern side of the ocean higher than that at the western side of the ocean at the same latitude. When the bathymetry reaches 500 m, the difference is only 3.5 kg/m^3^, and the difference becomes smaller and smaller as the bathymetry increases. There is little difference in the seawater density of the water layer below 500 m, and the difference is mainly concentrated in the high latitude area. This is because the downflow in this area transports the surface low-temperature seawater downward, making the density of seawater in the area near the poles higher than that in other areas at the same bathymetry. It can be observed from Figure 2 that seawater at a depth of 3000 m can still be affected by this process.

**Figure 3:**
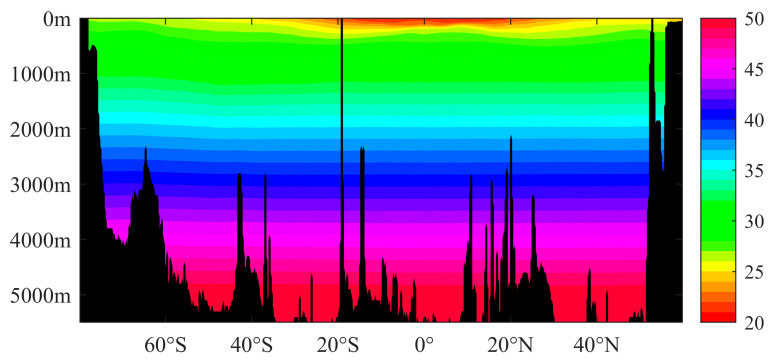
Density of seawater for the 170°W section of the Pacific Ocean (unit: kg/m^3^).

**Figure 4:**
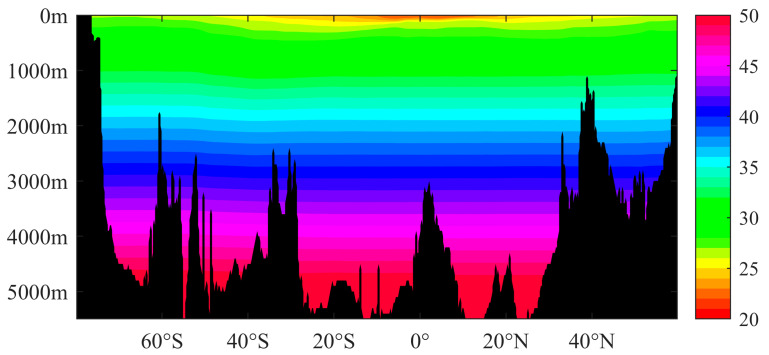
Density of seawater for the 30°W section of the Atlantic Ocean (unit: kg/m^3^).

In the vertical direction, the density of seawater increases with bathymetry from less than 1010 kg/m^3^ in the surface layer to 1050 kg/m^3^ at 5000 m (Table 1). For deeper waters in areas such as the Mariana Trench, the density will be greater. It can be observed from Figure 3 and Figure 4 that the density of upper seawater in the middle and low latitudes of the Pacific Ocean and Atlantic Ocean decreases sharply with the increase in bathymetry. This is because of the impact of solar radiation, which makes the temperatures in seawater in the middle and low latitude regions higher; however, solar radiation can only penetrate the shallow water layers, thus making the temperature drop sharply with the increase in bathymetry, and where the temperature changes less in the deep water. Therefore, the vertical gradient of the temperature in the upper layers is large, which leads to a large vertical gradient in density for shallow water in the middle and low latitudes, thus forming a thermocline. In the high latitude area, the annual low temperature, and the small difference in the surface water, lead to no obvious thermocline being formed, and where the density of seawater basically changes with pressure [22]. When the depth increases to several hundred meters, the seawater temperature basically remains unchanged, and the density of seawater is only affected by the pressure, which increases steadily with bathymetry.

## 4. Model of Seawater Density

To facilitate the calculation of the density of seawater by substitution into the relevant formula, it is necessary to express the distribution of the density of seawater as a functional expression. Gladkikh described the changes in density as a function of the bathymetry (consideration of density changes due to pressure) and the latitude (to explain density changes due to salinity and temperature) [20]:

(1) $$\rho \left(D,\varphi \right)=1000.0+\alpha \left(\varphi \right)+\beta \left(\varphi \right)D^{\vartheta \left(\varphi \right)}$$

where φ is the geographic latitude in the units of angle, and the parameters α, β, and ϑ are functions related to the latitude, which are defined as follows:

(2) $$\alpha \left(\varphi \right)=27.91-2.06exp\left[-{\left(0.0161\left|\varphi \right|\right)}^{5}\right]$$

(3) $$\beta \left(\varphi \right)=0.00637+0.00828exp\left[-{\left(0.017\left|\varphi \right|\right)}^{4.66}\right]$$

(4) $$\;\;\vartheta \left(\varphi \right)=0.964-0.091\;exp\;\left[-{\left(0.016\left|\varphi \right|\right)}^{5}\right]\;$$

The second item on the right side of the equal sign describes the density of seawater on the sea surface as increasing with the increase in latitude, and the third item on the right side includes the change with the bathymetry. The density increases nonlinearly with bathymetry, given that ϑ<1. It can be calculated that the density of seawater on the equatorial sea surface (bathymetry is 0; geographic latitude is 0) is 1027.91 kg/m^3^.

To improve the accuracy of the density model in shallow water, a correction of the pycnocline was introduced, namely:

(5) $$\rho \left(D,\varphi \right)=1000+\alpha \left(\varphi \right)\left\{\mu \left(\varphi \right)+\frac{1-\mu \left(\varphi \right)}{2}\left[1+\tanh\left(0.00988D-1.01613\right)\right]\right\}+\beta \left(\varphi \right)D^{\vartheta \left(\varphi \right)}$$

The parameter μ related to latitude is defined as:

(6) μ(φ)=0.928−0.079cos0.053φ

When the bathymetry is 0, the correction of the thermocline in seawater for the density model becomes:

(7) $$\rho \left(\varphi \right)=1000+\alpha \left(\varphi \right)\left[\mu +\frac{1-\mu }{2}\left(1-tanh1.01613\right)\right]$$

According to the description of Gladkikh et al., the accuracy of the density of shallow seawater was significantly improved after correction of the thermocline, and the relative accuracy was increased from 0.45% to 0.25% [20].

## 5. The Effect of the Anomaly of the Seawater Density on the Gravity Gradient

**Figure 5:**
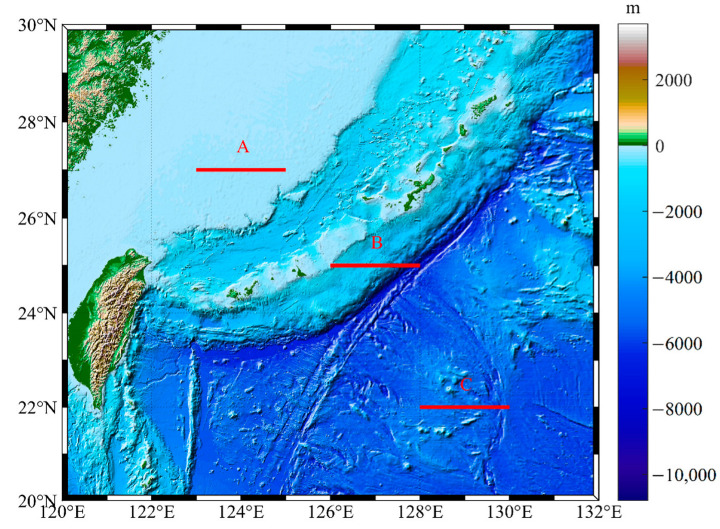
The topography and bathymetry to the east of Taiwan (the red lines in the figure show three profiles that were investigated: A, shallow water; B, large bathymetry change; C, deep water with small bathymetry change).

The area as shown in Figure 5 was selected, and the seawater density model of Gladkikh was used to approximate the seawater density of three tracks to calculate the contribution of the anomaly of the seawater density to the gravity gradient.

A local rectangular coordinate system was established for the mean sea level with the *x*, *y*, and *z* axes pointing to the east, north, and the upward direction, respectively. The constant density rectangular prism method is a popular tool for gravity gradient forward modeling. Therefore, according to basic principles, the area of interest was divided into multiple rectangular prisms in the horizontal direction according to the division of the grids of the longitude and latitude for the terrain data. The density of each rectangular prism was constant, and the length and width of the prism were determined as the average length and average width of all the grids in the study area. The constant density right rectangular prism method can be used to calculate the contribution of the gravity gradient of each prism with Equation (8), and then the gravity gradient at a certain location can be summed. However, the density of seawater changes with depth, so each right rectangular prism divided in the horizontal direction needs to be cut into multiple right rectangular prisms in the vertical direction. Because the density of the upper seawater changes dramatically, while the deep seawater changes slowly, it is necessary to layer the seawater unevenly. The seawater was set as 20 m for each layer within 200 m, and 200 m for each layer below 200 m. The density of seawater in each layer was replaced by the density at the average bathymetry of the seawater in this layer, and the horizontal position of the calculation point was located at the center of each grid.

The formula for calculating the gravity gradient of the *i*th prism at point ($x_{1}$, $x_{2}$, $x_{3}$) is:

(8) $$\Gamma_{jk}^{i}=G\int_{d_{i}}^{d_{i+1}}\int_{b_{i}-\frac{\Delta x_{2}}{2}}^{b_{i}+\frac{\Delta x_{2}}{2}}\int_{a_{i}-\frac{\Delta x_{1}}{2}}^{a_{i}+\frac{\Delta x_{1}}{2}}\frac{\partial^{2}}{\partial x_{j}\partial x_{k}}\frac{1}{r}\rho_{i}dx_{1}^{'}dx_{2}^{'}dx_{3}^{'}$$

where $G=6.674\times 10^{-11}m^{3}{\mathrm{kg}}^{-1}s^{-2}$ is Newton’s universal gravitational constant; $\rho_{i}$ is the seawater density of the ith prism, calculated by the seawater density model; $d_{i}$ and $d_{i+1}$ are the upper and lower standard layer bathymetry of the ith prism; ($a_{i}$, $b_{i}$) is the horizontal coordinate of the ith prism center point; $\Delta x_{1}$ and $\Delta x_{2}$ are the average length and average width of each grid in the study area, respectively. It was calculated that $\Delta x_{1}$ and $\Delta x_{2}$ in this area were 420 m and 461 m, respectively. $r=\sqrt{{\left(x_{1}-x_{1}^{'}\right)}^{2}+{\left(x_{2}-x_{2}^{'}\right)}^{2}+{\left(x_{3}-x_{3}^{'}\right)}^{2}}$ is the distance between the computation point ($x_{1}$, $x_{2}$, $x_{3}$) and the element point ($x_{1}^{'}$, $x_{2}^{'}$, $x_{3}^{'}$). *j* and *k* refer to the subscripts of *x*, which can be 1, 2 and 3, representing east, north and up, respectively. The gravity gradient at each grid point ($x_{1}$, $x_{2}$, $x_{3}$) is calculated by traversing, and the contribution of each grid is summed to obtain the gravity gradient at that location. 

To assess the balance between the efficiency of calculation and the error due to calculation, it is necessary to determine the scope of the calculation area. According to previous studies, if the rough terrain with a height variation of several thousand meters is used for forward modeling, the calculation area needs to reach 30′ × 30′, so that the gravity gradient obtained by forward modeling can reach an accuracy of 1E [1,23]. Thus, we use 121 × 121 terrain data grids with a resolution of 15′′ × 15′′ to calculate the gravity gradient in the central grid, and then traverse all grids in the study area to obtain the gravity gradient component values on the profile. We then calculate the gravity gradient caused by the mass deficiency of seawater along the profile ($\Gamma_{0}$ is calculated by using a constant seawater density $\rho_{0}=1030\;\mathrm{kg}/m^{3}$; $\Gamma_{1}$ is calculated by using the seawater density model $\rho_{1}$ in Formula (1); $\Gamma_{2}$ is calculated using the thermocline-corrected seawater density model $\rho_{2}$ in Formula (5)).

**Figure 6:**
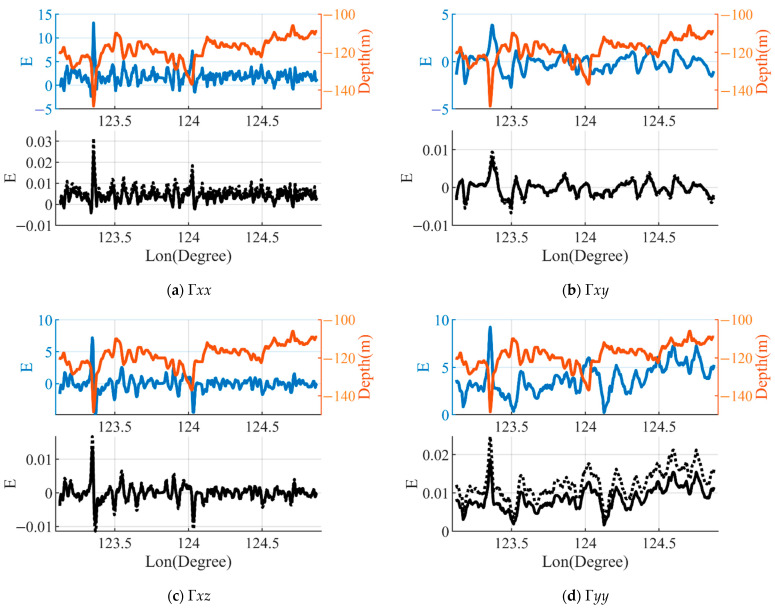

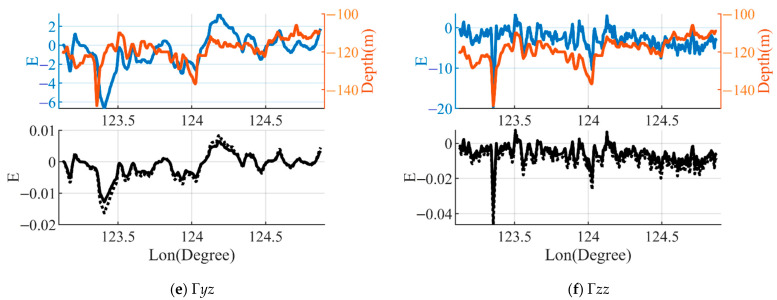
The bathymetry along the profile A and the $\Gamma_{0}$ calculated by using the constant seawater density $\rho_{0}$. The black solid line shows the effect of the error of the gravity gradient $\Delta \Gamma_{1}=\Gamma_{1}-\Gamma_{0}$ calculated using the seawater density model $\rho_{1}$. The black dashed line shows the effect of the error of the gravity gradient effect on $\Delta \Gamma_{2}=\Gamma_{2}-\Gamma_{0}$ calculated using the thermocline-corrected seawater density model $\rho_{2}$.

The first study area was located on the continental shelf of the East China Sea, and had a shallow water depth and gentle slope. The bathymetry ranged from 106 m to 148 m with an average bathymetry of 119 m. The results for the gravity gradient components are shown in Figure 6. It can be observed that the magnitude and range of variation for each component of the gravity gradient generated by the mass deficiency of seawater in this area are small; the maximum amplitude was about 20E and was related to the bathymetry, and the maximum range of the variation was less than 25E. The error in the gravity gradient caused by the differences in the density of seawater was small, with each component being less than 0.05E, which is closely related to the effect of the gravity gradient. The amplitude of the error obtained after the correction of the thermocline was larger than that of the uncorrected seawater density model, and the difference between the two errors of Γ*yy* was more apparent than that of the other components. The reason for this is that the thermocline-corrected seawater density model contains the latitude related parameter μ, which is in the y-direction.

**Figure 7:**
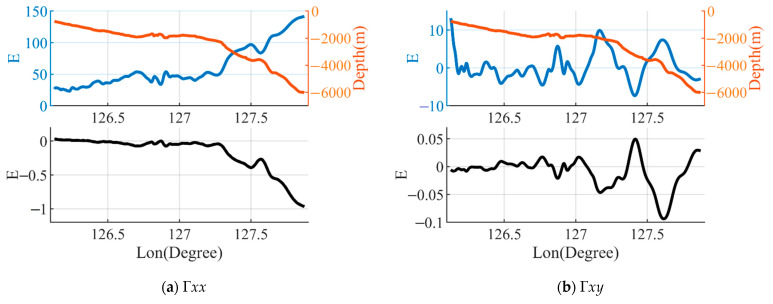

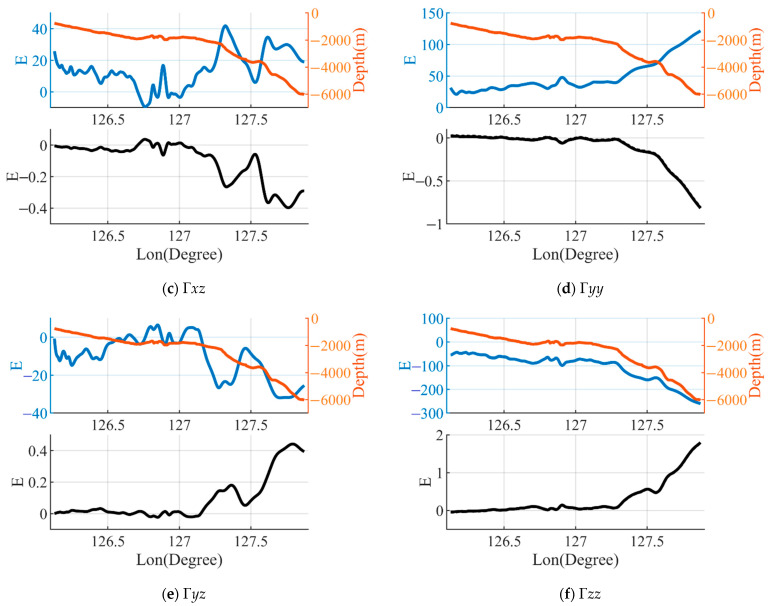
The bathymetry along the B profile and the $\Gamma_{0}$ calculated by using a constant seawater density $\rho_{0}$. The black solid line shows the effect of the error on the gravity gradient where $\Delta \Gamma_{1}=\Gamma_{1}-\Gamma_{0}$ was calculated by using the seawater density model $\rho_{1}$. The blackdashed line shows the effect of the error of the gravity gradient, where $\Delta \Gamma_{2}=\Gamma_{2}-\Gamma_{0}$ was calculated by using seawater density model $\rho_{2}$ based on the corrected thermocline.

The second study area was located near the Ryukyu Trench adjacent to the Miyako Strait on the continental slope. The bathymetry ranges from 753 m to 6043 m with an average depth of 2467 m. The results for the gravity gradient components are shown in Figure 7. It can be observed that the magnitude of each component of the gravity gradient generated by the mass deficiency in the seawater for this area is large, the range of variation being about 200E and the maximum amplitude reaching about 260E, which is related to the bathymetry. The amplitudes of Γ*xx*, Γ*yy* and Γ*zz* are significantly larger than those of the other three components and they are highly correlated with the change of bathymetry (the gravity gradient is smaller in shallow water and greater in deeper water). The Γ*xx*, Γ*yy* and Γ*zz* near the Ryukyu Trench can reach 140E, 120E and −260E, respectively. The error in the gravity gradient caused by the differences in the density of seawater is highly related to the effect of the gravity gradient. The errors of Γ*xx*, Γ*yy* and Γ*zz* near the Ryukyu Trench reach a maximum, the values being 1E, 0.8E and 1.8E, respectively. The amplitude of the error obtained after correction of the thermocline in this area is not much different from that of the uncorrected seawater density model.

**Figure 8:**
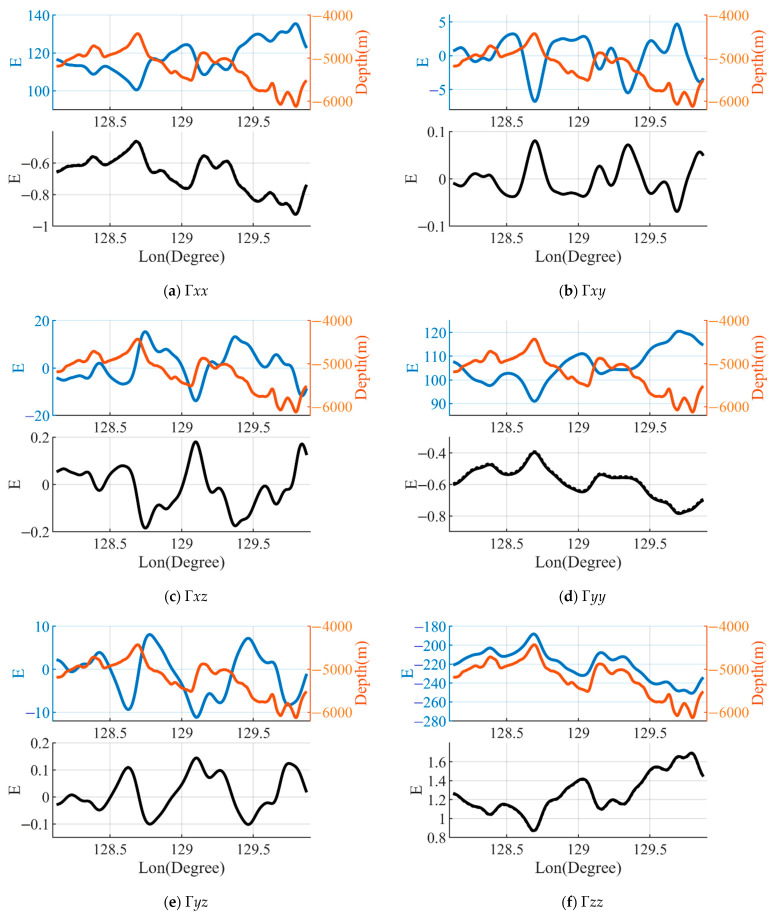
The bathymetry along the profile C and the $\Gamma_{0}$ calculated by using a constant seawater density $\rho_{0}$. The black solid line shows the effect of the error on the gravity gradient $\Delta \Gamma_{1}=\Gamma_{1}-\Gamma_{0}$ calculated by using the seawater density model $\rho_{1}$. The black dashed line shows the effect of the error of the gravity gradient $\Delta \Gamma_{2}=\Gamma_{2}-\Gamma_{0}$ calculated by using the corrected thermocline seawater density model $\rho_{2}$.

The third study area was located in the Philippine Basin, which has a large depth and a small slope. The bathymetry ranges from 4430 m to 6122 m with an average depth of 5204 m. The results for the gravity gradient components are presented in Figure 8. It can be observed that the magnitude for each component of the gravity gradient caused by the mass deficiency of the seawater in this area is large and the range of variation is small, the maximum amplitude reaching about 250E and the maximum range of variation being about 60E. As with the second profile, the amplitudes of Γ*xx*, Γ*yy* and Γ*zz* are significantly larger than the other three components and are highly correlated with the change in the bathymetry. The error of the gravity gradient caused by the difference in the density of seawater is highly related to the effect of the gravity gradient, the maximum error of Γ*zz* being 1.7E. The error in the amplitude obtained after correction of the thermocline in this area shows little difference compared to that for the uncorrected seawater density model.

According to the three profiles (Table 2), the values of Γ*xx*, Γ*yy* and Γ*zz* for profile B and profile C are significantly higher than those of profile A, and the values of Γ*xy*, Γ*xz* and Γ*yz* for profile B are significantly higher than those of profile A and profile C. These findings indicate that the values of Γ*xx*, Γ*yy* and Γ*zz* caused by the mass deficiencies in seawater are related to the bathymetry (the greater the bathymetry, the greater the gravity gradient), whereas the values of Γ*xy*, Γ*xz* and Γ*yz* are related to the slope (the greater the slope, the greater the value). The error in the gravity gradient caused by the difference in the seawater density is highly related to the effect of the gravity gradient. The values of Γ*xx*, Γ*yy* and Γ*zz* increase with the bathymetry, making the error in the gravity gradient due to the difference in the density of seawater greater. It is calculated that when the bathymetry reaches about 5000 m, the error of Γ*zz* can reach 1E, reflecting the fact that the density of seawater increases with the bathymetry. The difference between the actual seawater density and the constant value for the seawater density becomes larger for the increase in the bathymetry. The amplitude of the error of the gravity gradient obtained after correction of the thermocline results in little difference compared to that for the uncorrected seawater density model; however, the difference between the two errors in Γ*yy* is more obvious than that in the other components. The reason for this is that the thermocline-corrected seawater density model incorporates the latitude related parameter μ, which is in the y-direction. 

## 6. Conclusions

In this study, the distribution of the global seawater density was obtained by processing the seawater density data of the WOA18 dataset, which indicated differences in the density of seawater. The seawater density model proposed by Gladkikh et al. (2011). provided a more realistic representation of the seawater density than that of the constant seawater density model as used in traditional marine geophysics. The model proposed by Gladkikh et al. was used to calculate the gravity gradient, which was then used to quantify the error in the gravity gradient caused by using seawater data of constant density. The results demonstrated that the error in the gravity gradient was closely related to the effect of the gravity gradient. Calculations revealed that when the bathymetry reached about 5000 m, the error of Γ*zz* can reach 1E. The amplitude of the error obtained after correction of the thermocline was very small compared with the amplitude of the error for the uncorrected seawater density model. If a reference map of the gravity gradient with accuracy higher than 1E is to be prepared, the density of seawater cannot be regarded as a constant during forward modeling.

## Figures and Tables

**Table 1:** Statistics for the density of seawater at different depths in the global ocean (unit: kg/m^3^) (excluding marginal seas with density anomalies such as the Black Sea).

Depth	Max	Min	Mean	Std	Range
0 m	28.58	0.54	24.84	2.14	28.04
100 m	29.53	3.78	26.47	1.41	25.74
200 m	30.31	6.17	27.59	0.87	24.14
500 m	31.59	28.05	29.50	0.50	3.54
1000 m	33.96	30.92	32.18	0.33	3.04
2000 m	37.94	35.47	37.11	0.19	2.48
3000 m	42.55	39.82	41.75	0.18	2.74
5000 m	51.00	48.74	50.64	0.13	2.26

**Table 2:** The bathymetry and gravity gradient along the three profiles.

Profile	Bathymetry Range/m	Average Bathymetry/m	Maximum of Gravity Gradient/E	Range of Variation of Gravity Gradient/E	Maximum Error of Gravity Gradient Caused by Difference of Seawater Density/E
A	42	119	20	25	0.05
B	5290	2467	260	200	1.8
C	1692	5204	250	60	1.7

## Data Availability

Publicly available datasets were analyzed in this study. The seawater density data can be found here: https://www.ncei.noaa.gov/access/world-ocean-atlas-2018/ (accessed on 8 June 2022). The bathymetric data can be found here: https://www.gebco.net/data_and_products/gridded_bathymetry_data/ (accessed on 8 September 2021).

## References

[1] Zhu L. (2007). Gradient Modeling with Gravity and DEM. Ph.D. Thesis.

[2] Wu L., Tian X., Ma J., Tian J. (2010). Underwater object detection based on gravity gradient. IEEE Geosci. Remote. Sens. Lett..

[3] Zhu L., Jekeli C. (2009). Gravity gradient modeling using gravity and DEM. J. Geod..

[4] Gobashy M., Abdelazeem M., Abdrabou M., Khalil M.H. (2021). A hybrid PCG-bat algorithm for 2D gravity inversion: Applications for ore deposits exploration and interpretation of sedimentary basins. Ore Geol. Rev..

[5] Yang T. (2011). Simulation for Underwater Navigation System Based on Gravity Gradient. Ph.D. Thesis.

[6] Liu F., Qian D., Li Y., Zhang Y. (2010). Influences of density error on gravity gradient forward results. J. Huazhong Univ. Sci. Technol..

[7] Yan Z., Ma J., Xu M., Yang F., Tian J., Fu W. (2014). Modeling gravity gradient maps using the isostasy models. Prog. Geophys..

[8] Yan Z. (2015). Gravity gradient for submarine threat object detection. Ph.D. Thesis.

[9] Karcol R. (2018). The gravitational potential and its derivatives of a right rectangular prism with depth-dependent density following an n-th degree polynomial. Stud Geophys. Geod..

[10] Tenzer R., Vajda P. (2008). Global map of the gravity anomaly corrected for complete effects of the topography, and of density contrasts of global ocean, ice, and sediments. Contrib. Geophys. Geod..

[11] Tenzer R., Vajda P. (2008). Global secondary indirect effects of topography, bathymetry, ice and sediments. Contrib. Geophys. Geod..

[12] Tenzer R., Hamayun K., Vajda P. (2009). Global maps of the CRUST 2.0 crustal components stripped gravity disturbances. J. Geophys. Res. Solid Earth.

[13] Tenzer R., Vajda P., Hamayun P. (2010). A mathematical model of the bathymetry-generated external gravitational field. Contrib. Geophys. Geod..

[14] Yang J. (2017). Seafloor Topography Estimation from Gravity Gradient. Ph.D. Thesis.

[15] Yang J., Jekeli C., Liu L. (2018). Seafloor topography estimation from gravity gradients using simulated annealing. J. Geophys. Res. Solid Earth.

[16] Wang W. (2009). Underwater Navigation Methods Based on Gravity and Environmental Features. Ph.D. Thesis.

[17] Xian P., Ji B., Bian S., Liu B. (2022). Influence of sea level anomaly on underwater gravity gradient Measurements. Sensors.

[18] Zweng M.M., Seidov D., Boyer T.P., Locarnini M., Garcia H.E., Mishonov A.V., Baranova O.K., Weathers K., Paver C.R., Smolyar I., Mishonov A. (2019). Salinity. World Ocean Atlas 2018.

[19] Locarnini R.A., Boyer T.P., Mishonov A.V., Reagan J.R., Zweng M.M., Baranova O.K., Garcia H.E., Seidov D., Weathers K.W., Paver C.R., Mishonov A. (2019). Density. World Ocean Atlas 2018.

[20] Vladislav G., Robert T. (2011). A Mathematical model of the global ocean saltwater density distribution. Pure Appl. Geophys..

[21] Feng S., Li F., Li S. (1999). An Introduction to Marine Science.

[22] Talley L.D., Pickard G.L., Emery W.J., Swift J.H., Zhang H. (2019). Descriptive Physical Oceanography: An Introduction.

[23] Jekeli C., Zhu L. (2006). Comparison of methods to model the gravitational gradients from topographic data bases. Geophys. J. Int..

